# Novel Multiple Markers to Distinguish Melanoma from Dysplastic Nevi

**DOI:** 10.1371/journal.pone.0045037

**Published:** 2012-09-27

**Authors:** Guohong Zhang, Gang Li

**Affiliations:** 1 Department of Dermatology and Skin Science, Jack Bell Research Centre, Vancouver Coastal Health Research Institute, University of British Columbia, Vancouver, British Columbia, Canada; 2 Department of Pathology, Shantou University Medical College, Shantou, Guangdong, China; The University of Queensland, Australia

## Abstract

**Background:**

Distinguishing melanoma from dysplastic n**e**vi can be challenging.

**Objective:**

To assess which putative molecular biomarkers can be optimally combined to aid in the clinical diagnosis of melanoma from dysplastic nevi.

**Methods:**

Immunohistochemical expressions of 12 promising biomarkers (pAkt, Bim, BRG1, BRMS1, CTHRC1, Cul1, ING4, MCL1, NQO1, SKP2, SNF5 and SOX4) were studied in 122 melanomas and 33 dysplastic nevi on tissue microarrays. The expression difference between melanoma and dysplastic nevi was performed by univariate and multiple logistic regression analysis, diagnostic accuracy of single marker and optimal combinations were performed by receiver operating characteristic (ROC) curve and artificial neural network (ANN) analysis. Classification and regression tree (CART) was used to examine markers simultaneous optimizing the accuracy of melanoma. Ten-fold cross-validation was analyzed for estimating generalization error for classification.

**Results:**

Four (Bim, BRG1, Cul1 and ING4) of 12 markers were significantly differentially expressed in melanoma compared with dysplastic nevi by both univariate and multiple logistic regression analysis (p < 0.01). These four combined markers achieved 94.3% sensitivity, 81.8% specificity and attained 84.3% area under the ROC curve (AUC) and the ANN classified accuracy with training of 83.2% and testing of 81.2% for distinguishing melanoma from dysplastic nevi. The classification trees identified ING4, Cul1 and BRG1 were the most important classification parameters in ranking top-performing biomarkers with cross-validation error of 0.03.

**Conclusions:**

The multiple biomarkers ING4, Cul1, BRG1 and Bim described here can aid in the discrimination of melanoma from dysplastic nevi and provide a new insight to help clinicians recognize melanoma.

## Introduction

Malignant melanoma is one of the most aggressive malignancies in humans with an estimated total of 48,000 fatalities worldwide per year, and the incidence of melanoma continues to rise [1]. Since melanoma is very resistant to conventional chemotherapy with only 14 percent of patients with metastatic melanoma survive for 5 years [2], it accounts for almost 75% of deaths related to skin cancer.

The accuracy of melanoma diagnosis is critical for the containment of the malignancy and the stage at diagnosis is a significant factor related to melanoma-specific survival [3]. If melanoma patients were misdiagnosed they might be inadequately treated and potentially be at risk for regional or systemic spread [4]. However, distinguishing malignant melanoma from dysplastic nevi remained problematic due to the wide variation in morphologic features. Dysplastic nevi often have some clinical and histologic features that overlap with melanoma, such as an irregular border, the asymmetric distribution of pigmentation, cytologic atypia and dermal inflammatory response [5]–[7]. The clinical picture, dermatoscopy and molecular or genetic testing often cannot distinguish a histological dysplastic nevus from a melanoma [8]. Therefore, a subset of melanomas may easily be misdiagnosed to be dysplastic or Spitz nevi [9], [10]. One of the reasons for clinical misdiagnosis is that distinctive biomarkers between melanoma and dysplastic nevi are absent.

So far, despite S100 immunostaining with the 97–100% sensitivity and 75–87% specificity, additional higher specificity markers such as HMB-45 (69–93%), and MART-1/Melan-A (75–92%) have been used to assist in the differential diagnosis for melanoma [11]. However, melanocytic lesions including dysplastic nevi also stain positive for S100, MART-1/Melan-A and HMB-45 [12], [13]. These markers were not useful in separating melanoma from dysplastic nevi [14]. In additional, there were subsets of melanoma cases without staining for S100 protein, HMB-45, and MART-1/Melan-A [15]. To overcome these limitations, there is now a strong rationale to add more specific immunohistochemical markers to reliably distinguish melanoma from dysplastic nevi. Furthermore, artificial neural network (ANN) and classification tree methods offer comprehensive model to find and verify predictor variables to improve overall specificity and sensitivity to discriminate melanoma from dysplastic nevi. The ANN is a powerful computational tool imitating human neuronal systems, and it has shown ability to determine complex relationships between variables with high predictive accuracies on blind data [16]. Classification tree presents a decision tree, which is intuitive and facilitates the allocation of patients into subgroups by following the flow-chart form that is simple to interpret and may be applied at the bedside [17].

As the diagnosis of melanoma can be challenging sometimes, the application of melanoma tissue microarray (TMA) datasets may lead to the identification of previously unrecognized markers. Our previous studies found that 12 markers including pAkt, Bim, BRG1, BRMS1, CTHRC1, Cul1, ING4, MCL1, NQO1, SKP2, SNF5 and SOX4 were associated with melanoma progression [18]–[29]. In this present study, we attempted to assess these 12 putative markers to identify which can be optimally combined to aid in the discrimination of melanoma from dysplastic nevi.

## Materials and Methods

### Study Population and Tissue Microarrays

Formalin-fixed and paraffin-embedded biopsies were obtained from the 1990–1998 archives of the Department of Pathology, Vancouver General Hospital. TMA were constructed as previously described [18]. Briefly, using core diameters of 0.6 mm taken from the paraffin blocks, assembled using a tissue-array instrument (Beecher Instruments, Silver Spring, MD), and cut with a Leica microtome (Leica Microsystems Inc, Bannockburn, IL). The tissue microarray consisted of 51 dysplastic nevi, 74 primary melanomas without distant metastasis, and 48 metastatic melanomas, including 20 were obtained from lymph nodes and 28 from other metastatic organs. For 122 melanomas, 28 melanomas were located in sun-exposed sites (head and neck), and 94 were located in sun-protected sites (other locations). In the 74 primary melanoma, there were 44 male and 30 female, with age ranging from 21 to 93 years (median, 60 years), and for 48 cases of metastatic melanomas, there were 34 male and 14 female, with age ranging from 27 to 89 years (median, 59 years). The use of human skin tissues and the waiver of patient consent in this study were approved by the Clinical Research Ethics Board of the University of British Columbia.

### Immunohistochemistry

TMA slides were baked at 55°C for 30 minutes and dewaxed with three consequent 5-min washes with xylene. Then the rehydrated tissues were performed by a series of 5-min washes in 100%, 95%, and 80% ethanol, followed by two washes in distilled water. Antigen retrieval was performed by heating the samples at 95°C for 30 minutes in 10 mM sodium citrate (pH 6.0). After inactivating the endogenous peroxidase by incubating in 3% hydrogen peroxide for 30 minutes and blocking with universal blocking serum for 30 minutes (Dako Diagnostics, Carpinteria, CA), then TMA slides were incubated with primary antibody [rabbit polyclonal anti-pAkt (1∶100 dilution; Cell Signaling Technology, Beverly, MA); rabbit polyclonal anti-Bim (1∶50 dilution; NeoMarkers, Fremont, CA); rabbit polyclonal anti-BRG1 (1∶100 dilution; Santa Cruz Biotechnology, Santa Cruz, CA); rabbit polyclonal anti-CTHRC1 (4 µg/mL final concentration; Immunochem Pharmaceutical Inc, Burnaby, BC, Canada); mouse monoclonal anti-BRMS1 (1∶200 dilution; provided by Dr Danny R. Welch, University of Alabama at Birmingham); rabbit polyclonal anti-ING4 (1∶50 dilution; ProteinTech Group, Chicago, IL); mouse monoclonal anti-MCL1 (1∶100 dilution; Santa Cruz); mouse monoclonal anti-NQO1 (1∶100 dilution; Santa Cruz ); mouse monoclonal anti-SNF5 (1∶200 dilution; Abcam, Cambridge, MA); mouse monoclonal anti-Cul1(1∶100 dilution; Santa Cruz); mouse monoclonal anti-SKP2 (1∶100 dilution; clone A-2; Santa Cruz); and rabbit polyclonal anti-SOX4 (1∶25 dilution; Abcam) at 4°C overnight. The TMA slides were then incubated for 30 min each with a biotin-labeled secondary antibody and then streptavidin-peroxidase (Dako Diagnostics). The samples were developed using 3,3-diaminobenzidine substrate (Vector Laboratories, Burlington, Ontario, Canada) and counterstained with hematoxylin. Dehydration was then performed following a standard procedure and the slides were sealed with coverslips. The technical negative control used for Immunohistochemistry included the use of PBS instead of primary antibody, with all other conditions kept the same.

### Evaluation of Immunohistochemical Staining

The evaluation of staining was blindly and independently examined by three observers, including one dermatopathologist. Staining intensity was graded using the following scale: no staining (0), weak (1), moderate (2), and strong (3). The percentage of positive cells was also scored into 4 categories: 1 (0–25%), 2 (26–50%), 3 (51–75%), and 4 (76–100%). The level of staining was evaluated by immunoreactive score (IRS), which is calculated by multiplying the scores of staining intensity and the percentage of positive cells. Based on the IRS, staining pattern was defined as 0-negative, 1-weak (IRS 1–4), 2-moderate (IRS 6–9) and 3-strong (IRS 12). Consensus score was determined for any discrepant scoring for each marker.

### Calculating the Index Score for Multiple Biomarkers

To assess the value of the multiple biomarkers, the index score was calculated for the 4 biomarkers, Bim, BRG1, Cul1 and ING4 using permutation and combination method for effective in data fusion [30]; combined rank scores 0–7 of 2 markers, ING4-Cul1; 0–17 of 3 markers, ING4-Cul1-BRG1; 0–30 of 4 markers, ING4-Cul1-BRG1-Bim.

### Statistical Analysis

The likelihood of prediction for each marker’s score was assessed with univariate logistic regression and multivariate logistic regression was performed on significant markers (p<0.05) from univariate logistic regression with optimal scale partitioning [31]. For the univariate logistic regression, negative staining was set as the reference category, weak, moderate, strong and these combinations were compared with negative category, and the overall p value was used to determine the significance. The specificity and sensitivity for individual marker and combination of 2-markers, 3-markers and 4-markers were analyzed using and receiver operating characteristic (ROC) curves and by calculating the area under the ROC curve (AUC). The ANN, following training, could discriminate important patterns in input and respond with an appropriate output, and when the ANN was trained and tested after optimizing the input parameters, the overall predictive accuracy can be obtained. In this study, the ANN was performed by the Radial Basis Function Algorithm (RBFA), consisting of 12 units in input layers of covariates plus the number of factor levels, automatically calculating number of units in hidden layer within 1 to 50 and 2 out-put layers (dysplastic nevi and melanoma). The number of nodes was determined by trial and error to produce the best performance. Classification tree was constructed by the classification and regression tree (CART) model to examine markers simultaneous optimizing the accuracy of melanoma. The decision trees depicting the classification rules generated through recursive partitioning. When growing each tree, we assigned equal prior probabilities to the normal and cancer cohorts, and equal misclassification costs. To assess the amount of over-fitting, 10-fold cross-validation experiments was performed using the SE rule as described by Breiman et al [32]. In each of those 1,000 experiments, the data set was randomly split into 10 smaller data sets and a pruning method was used to choose the best number of nodes for the original tree pruned with respect to 90% of the data according to the misclassification rate for the other 10% of the data. All statistical tests were two-sided. Significance levels were set at p<0.05. All statistical analyses were carried out using the SPSS version 16.0 software (SPSS, Chicago, IL, USA).

## Results

### Four of 12 Markers Expressed Differently in Melanoma and Dysplastic Nevi

**Figure 1:**
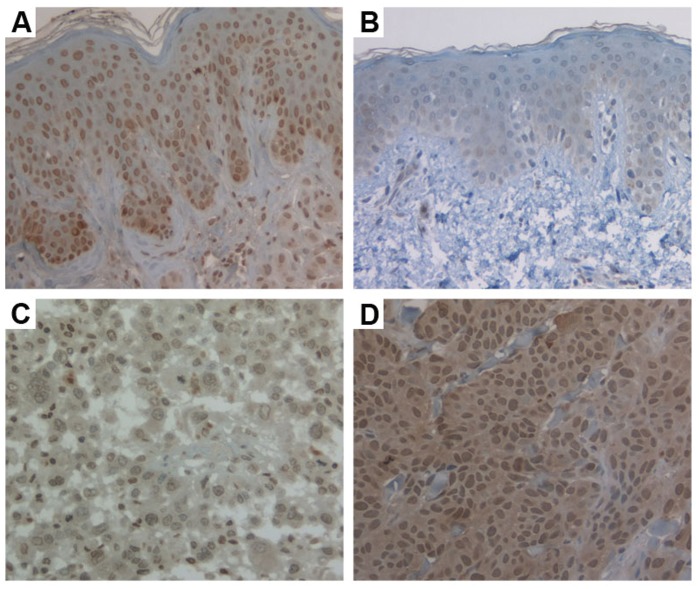
Representative images of immunochemistry staining of dysplastic nevi and melanoma. (a) Dysplastic nevi with strong BRMS1 staining; (b) Dysplastic nevi with weak Cul1 staining; (c) Melanoma with weak BRMS1 staining; (d) Melanoma with strong Cul1 staining. Magnification, ×200.

Due to loss of biopsy cores or insufficient tumor cells present in the cores, 33 cases of dysplastic nevi, 122 melanomas could be evaluated finally for staining of all 12 markers. Of 12 markers, 5 markers (pAkt, Bim, CTHRC, MCL1 and NQO1) had predominantly cytoplasmic staining, 4 markers (BRMS1, Cul1, ING4 and SOX4) had nuclear staining, and other 3 markers (BRG1, SKP2 and SNF5) contained both cytoplasmic and nuclear staining. Representative images of immunochemistry staining were illustrated in Figure 1 and Table 1 summarized the results of the staining for each marker.

**Table 1:** Biological functions of 12 markers.

Marker	Full name	Function	Localization
pAkt	Protein Kinase B	A serine/threonine kinase that leads to stimulation of cellcycle progression, cell proliferation, and inhibition of apoptosis	Cytoplasm
Bim	BCL2-like 11	A BH3-only protein belonging to the Bcl-2 family ofapoptotic regulators	Cytoplasm
BRG1	Brahma-related gene-1	A component of SWI ?SNF chromatin remodelling complex	NucleusCytoplasm
BRMS1	Breast cancer metastasis suppressor 1	A component of the mSin3a family of histone deacetylasecomplexes	Nucleus
CTHRC1	Collagen triple helix repeat containing 1	A pro-migratory protein first found to be expressedduring rat tissue repair process	Cytoplasm
Cul1	Cullin 1	A rigid scaffold in SCF (Skp1/Cullin/Rbx1/F-box protein)complex	Nucleus
ING4	Inhibitor of growth family, member 4	Tumor suppressor which interacts with p53, inhibits cell growth, and induces apoptosis	Nucleus
MCL1	Myeloid cell leukemia sequence 1 (BCL2-related)	Myeloid cell leukaemia-1, an anti-apoptotic protein	Cytoplasm
NQO1	NAD(P)H dehydrogenase, quinone 1	A key enzyme involved in metabolism of quinones	Cytoplasm
SKP2	S-phase kinase-associated protein 2	An F-box protein, targets cell cycle regulators viaubiquitin-mediated degradation	CytoplasmNucleus
SNF5	SWI/SNF related, matrix-associated, actin-dependent A regulator of chromatin, subfamilyb, member 1	Tumor suppressor, the core subunit of SWI/SNF complex	NucleusCytoplasm
SOX4	Sex determining region Y-box 4	Embryonic development and differentiation	Nucleus

Initially, each of the 12 markers was evaluated individually for its ability to predict melanoma from dysplastic nevi using univariate logistic regression analysis. The results demonstrated that the expression of Bim, BRG1, Cul1 and ING4 differed significantly between melanoma and dysplastic nevi, using the optimal scale partitioning of negative, weak to moderate and strong staining of these markers (Table 2). ING4 and Cul1 had the most significantly statistical differences between dysplastic nevi and melanoma.

**Table 2:** Discrimination of melanoma from dysplastic nevi using individual marker via univariate logistic regression analysis.

Marker	Optimal scale partitioning	Chi-square	P value
Akt	0 vs. 1–2 vs. 3	3.203	0.202
Bim	0 vs. 1–2 vs. 3	10.712	0.005
BRG1	0 vs. 1–2 vs. 3	9.595	0.008
BRMS1	0 vs. 1–2 vs. 3	0.893	0.640
CTHRC1	0 vs. 1–2 vs. 3	3.398	0.183
Cul1	0 vs. 1–2 vs. 3	30.991	1.961×10^−7^
ING4	0 vs. 1–2 vs. 3	21.218	2.469×10^−5^
MCL1	0 vs. 1–2 vs. 3	1.349	0.510
NQO1	0 vs. 1–2 vs. 3	5.714	0.057
SKP2	0 vs. 1–2 vs. 3	3.705	0.517
SNF5	0 vs. 1–2 vs. 3	0.788	0.674
SOX4	0 vs. 1–2 vs. 3	2.222	0.329

0, negative; 1, weak; 2, moderate; 3, strong.

The four markers (Bim, BRG1, Cul1 and ING4) were selected for confirmation based on statistically significant differences in univariate logistic regression analysis. Using the multiple logistic regression analysis, there is statistically significant difference in expression for 4 markers comparing melanoma with dysplastic nevi. Cul1 had the most significant statistical difference between dysplastic nevi and melanoma (Table 3). This provided directional confirmation of the results of the previous univariate logistic regression analysis.

**Table 3:** Discrimination of melanoma from dysplastic nevi via multiple logistic regressions.

Marker	Chi-square	P value
**Bim**	7.416	0.025
**BRG1**	10.723	0.005
**Cul1**	18.820	8.190×10^−5^
**ING4**	12.817	0.002

### Diagnostic Accuracy of Four Individual Markers for Melanoma

In order to test the diagnostic accuracy of these 4 markers, sensitivity, specificity and AUC were constructed for 4 individual markers with three levels of negative, weak to moderate and strong (Table 4), and the results revealed that ING4 and Cul1 had sensitivity of 93.44% and 90.98%, and specificity of 66.67% and 61.83%, respectively. BRG1 had the lowest specificity (26.67%) but it had highest sensitivity (97.14%).

**Table 4:** Diagnostic accuracy for melanoma via sensitivity and specificity of each individual marker.

Marker	Sensitivity, %	Specificity, %
Bim	81.06	45.46
BRG1	97.14	26.67
Cul1	90.98	61.83
ING4	93.44	66.67

### Diagnostic Accuracy of Different Optimal Combination

**Figure 2:**
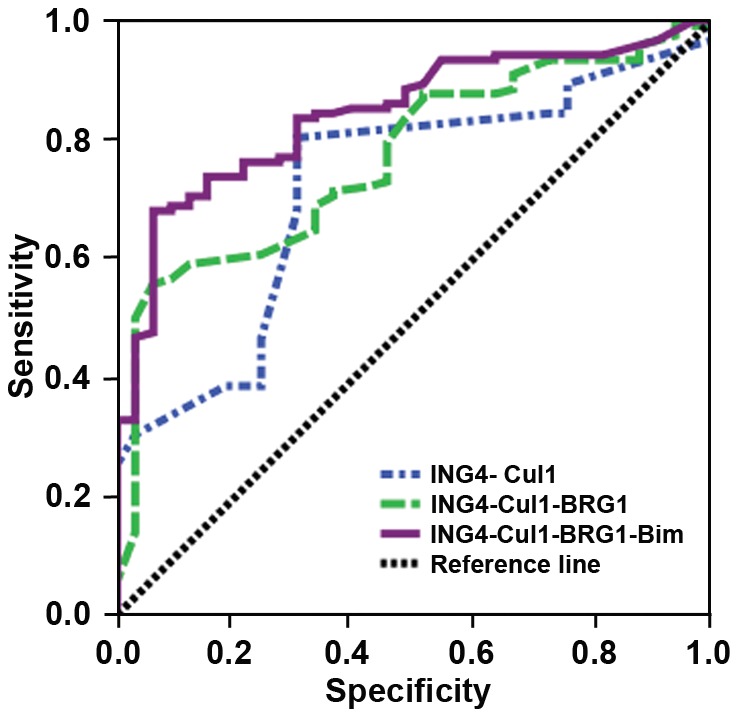
ROC curve for 4-markers (ING4-Cul1-BRG1-Bim, purple curve), 3-markers (ING4-Cul1- BRG1, green curve) and 2-marker (ING4-Cul1, blue curve).

The advantage of using a panel of markers was to improve sensitivity and specificity. In formulating multiple-marker strategies, we chose 2-markers (ING4-Cul1), 3-markers (ING4-Cul1-BRG1), and 4-markers (ING4-Cul1-BRG1-Bim) according to the specificity values. AUC is a global measure for characterizing utility, multiple-marker strategies resulted in improvement in the area under the ROC curve. Multiple-marker distinguishing capability can be significantly enhanced when compared to single-marker capability. Results showed that the multiple markers led to increased accuracy of melanoma diagnosis. A panel consisted of 4 markers (ING4-Cul1-BRG1-Bim) achieved 94.3% sensitivity and attained 81.8% specificity for discrimination of melanoma from dysplastic nevi, and AUC is 84.3%, higher than 71.9% for 2-markers (ING4-Cul1) and 76.9% for 3-markers (ING4-Cul1-BRG1) (Figure 2).

### Artificial Neural Network

**Figure 3:**
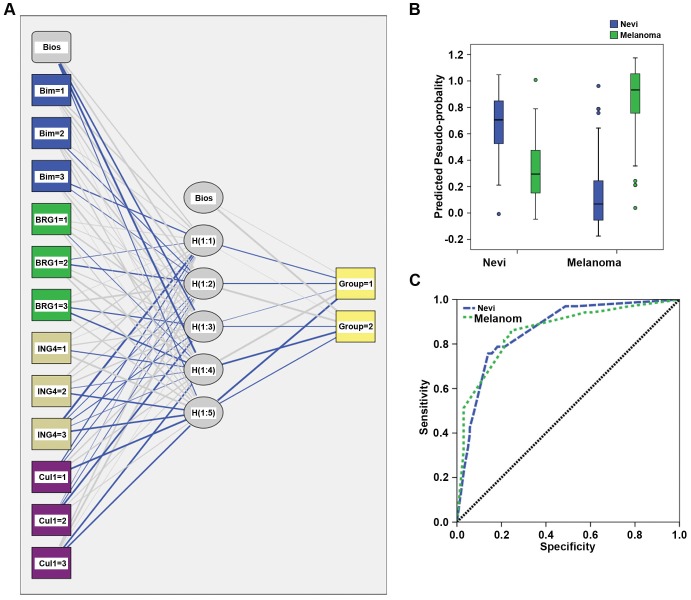
Architecture and performance of ANN. (a) ANN architecture. The network consisted of three layers: Input (boxes 1–12), hidden (circles 1–6) and output (group 1: dysplastic nevi, group 2: melanoma) layer, respectively. (b) ANN predicted-by-observed performance chart. The box plots represent the predicted-pseudo-probabilities for the output category; dysplastic nevi (blue) and melanoma (green) plotted against the known clinical status for dysplastic nevi and melanoma. (c) The ROC curve for dysplastic nevi and melanoma separately.

ROC curve results prompted us to generate a network classifier to confirm 4-marker differentiation between dysplastic nevi and melanoma. A separate output unit was created for dysplastic nevi and melanoma shown in network architecture (Figure 3a). The ANN correctly classified 83.2% of training and 81.2% of testing (Figure 3b). The AUC for ANN on prediction were 86.2% for dysplastic nevi and 86.0% for melanoma (Figure 3c). The overall performance of the ANN was consistent with the results obtained by ROC curve analysis.

### Construction of Classification Model for Four Markers

**Figure 4:**
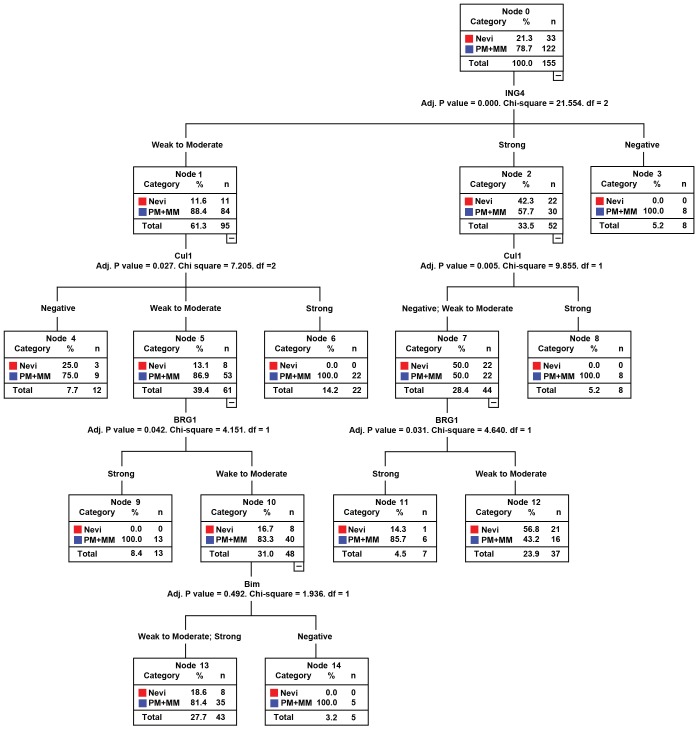
Classification tree of ING4, Cul1, BRG1 and Bim biomarkers for dysplastic nevi and melanoma. Nevi, dysplastic nevi; PM+MM, primary and metastatic melanoma.

We next determined the contribution of classification with the CART model by measuring the sensitivity of the classification to a change in the expression level of each marker. In this way, we ranked the markers according to their significance for the classification. We explored the utility of the multi-marker assay in diagnosing melanoma with top-to-bottom differences. ING4 was the first determinant or the initial node for classification tree (Figure 4). There was perfect separation of the 3 distributions of top-to-bottom difference scores between dysplastic nevi and melanomas, 8 of 155 melanomas lost ING4 expression, whereas 22 of 33 dysplastic nevi had strong expression. Application of this classification scheme resulted in a sensitivity of 86.9% and a specificity of 76.1%, and AUC of 82.9% in the diagnosis of melanoma. The mean accuracy and error of the classification tree estimated by performing 10-fold cross-validation was 79.2% and 0.03.

**Figure 5:**
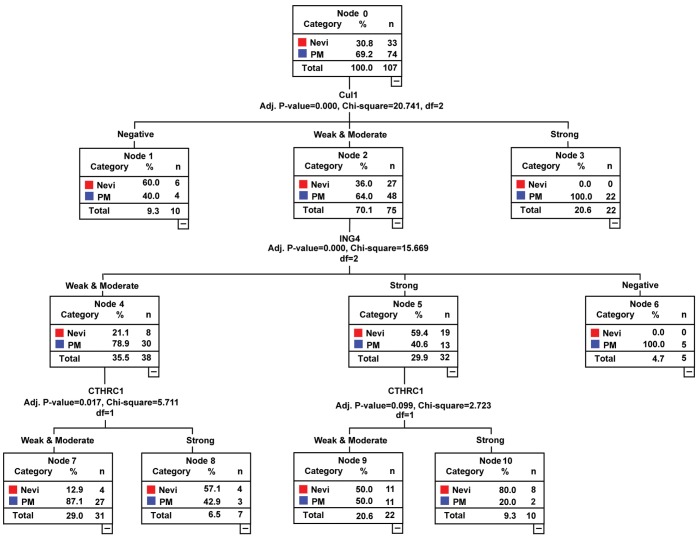
Classification tree of Cul1, ING4 and CTHRC1 biomarkers for dysplastic nevi and primary melanoma. Nevi, dysplastic nevi; PM, primary melanoma.

**Figure 6:**
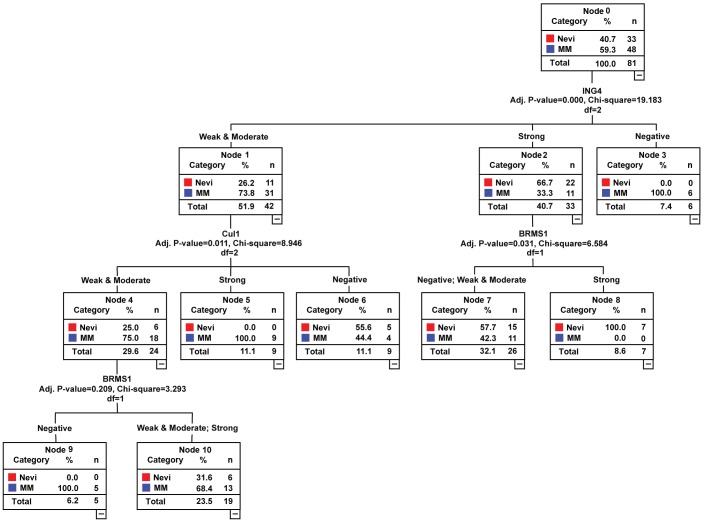
Classification tree for ING4, Cul1 and BRMS1 biomarkers for dysplastic nevi and metastatic melanoma. Nevi, dysplastic nevi; MM, metastatic melanoma.

After univariate and multivariate logistic regression analyses, four markers (BRG1, CTHRC1, Cul1 and ING4) with 91.2% sensitivity and 85.9% specificity, and five markers (Bim, BRG1, BRMS1, Cul1 and ING4) with 93.4% sensitivity and 86.1% specificity were obtained for distinguishing primary and metastatic melanoma from dysplastic nevi using ROC analysis, respectively. The classification tree results of primary and metastatic melanoma showed that ING4 and Cul1 were on the top of tree structure. Furthermore, CTHRC1 may be a useful marker for primary melanoma (Figure 5) and BRMS1 serves an important marker for metastatic melanoma distinguishing from dysplastic nevi (Figure 6).

## Discussion

Specific markers for discrimination of melanoma from dysplastic nevi were scarce. The misdiagnosis of melanoma is the second most common reason for cancer malpractice claims in the United States [33]. To overcome these limitations, we utilized TMA to evaluate diagnostic usefulness of biomarkers for distinguishing melanoma from dysplastic nevi. Our data demonstrated that 4 of 12 markers were diagnostically useful, either singly or in combination for distinguishing melanoma from dysplastic nevi. This method may represent a simple and easy way to implement the translation of tissue microarray data into clinical practice. Four markers described here could be used to assist in the histological diagnosis of melanoma, thereby providing important information to clinical pathologists.

We found that Bim, BRG1, Cul1 and ING4 were differently expressed between melanoma and dysplastic nevi using the univariate logistic regression (p < 0.01). Univariate analyses alone may not be the best approach in choosing which markers to combine in a predictive panel of markers. The differential expressions of Bim, BRG1, Cul1 and ING4 between melanoma and dysplastic nevi were confirmed by the multiple logistic regressions (p < 0.05). These results imply that it is possible to combine this 4-marker panel to distinguish melanoma from dysplastic nevi. For the single biomarker accuracy, ING4 has high sensitivity of 93.46% and specificity of 66.67%. We found that applying the multiple-biomarker strategies improved specificity and AUC. Combined 4-markers showed substantial improvement specificity over single marker, from the highest 66.67% to 81.8%, and AUC reached 84.3%. Our experience, as well as that of others, has shown that a panel of markers is most helpful for differentiating between melanoma and dysplastic nevi. Furthermore, the reliability of 4 markers to distinguish melanoma from dysplastic nevi was confirmed by ANN. ANN analysis as a statistical modeling tool has demonstrated the ability to assimilate information from multiple sources and detect subtle and complex patterns [34]. In our study, combination of 4 markers improved AUC prediction of 86.2% for dysplastic nevi and 86.0% for melanoma.

Utility of markers were measured by the classification tree, we identified ING4, Cul1 and BRG1 were the most important classification parameters in ranking top-performing biomarkers. Classification tree was separated by the most powerful prediction variable ING4, and the 10 fold of cross-validation error was 0.03, suggesting that the tree was stable and reliable. CART is an alternative to logistic regression and has several advantages as a tool for developing clinical decision rules. A decision tree, on the other hand, is easily understood by physicians. The most clinically useful information gained by using the prediction CART tree is that ING4 is priority marker, then Cul1 and BRG1 in clinical application. We analyzed primary and metastatic melanomas separately, the ING4 and Cul1 were the best in the classification tree. Furthermore, the CTHRC1 marker may be a useful marker for primary melanoma and BRMS1 serves an important marker for metastatic melanoma distinguishing form dysplastic nevi. The classification tree requires additional research to validate the diagnostic value of Bim, BRG1, Cul1 and ING4 in an independent data set.

Biological interpretation is required to understand why the proposed markers are significantly different and as a utilization in patients with melanoma and dysplastic nevi. Several of the markers incorporated into our study have been previously demonstrated to have a role in driving melanoma progression. ING4, a tumor suppressor, mediates chromatin modification and has a suppressive effect on tumorigenesis and innate immunity [35], [36]. It inhibits melanoma angiogenesis by suppressing NF-κB pathway and is involved in melanomagenesis and induces growth suppression and apoptosis in melanoma cell line [37], [38]. Cul1, a member of Cullin family, plays an important role in protein degradation and protein ubiquitination, is increased in early stages of human melanoma and promotes melanoma cell proliferation through regulating p27. [27], [39] BRG1, the catalytic subunit of the SWI/SNF chromatin remodelling complex, is a novel binding partner of the tumor suppressor p16INK4a, which is one of the most important melanoma susceptibility genes identified to date [40] and is involved in melanoma initiation [20]. Bim is a novel member of the Bcl-2 family that promotes apoptosis [41]. Among all the BH3-only protein members, Bim has been shown to have the ability to interact with all Bcl-2 members, suggesting that it may serve as a key factor in the event of apoptosis and thus inhibition of Bim function may be involved in tumorigenesis.

In summary, we describe a multi-marker immunohistochemical panel of Bim, BRG1, Cul1 and ING4 which may aid in differential diagnosis for melanoma from dysplastic nevi.
